# Disease surveillance data sharing for public health: the next ethical frontiers

**DOI:** 10.1186/s40504-018-0078-x

**Published:** 2018-07-04

**Authors:** Patty Kostkova

**Affiliations:** Institute for Risk and Disaster Reduction (IRDR), UCL, Gower Street, London, WC1E 6BT UK

## Abstract

In the recent years, we have been witnessing a digital revolution in public and global health creating unprecedented opportunities for epidemic intelligence and public health emergencies. However, these opportunities created a double edge sword as access to data, quality monitoring and assurance, as well as governance and regulation frameworks for data privacy are lagging behind technological achievements.

In this paper we identify three ethical challenges: sharing data across various early warning tools to support risk assessment. Secondly, define the challenges to be addressed by the legal frameworks for public health data sharing to unlock the potential of population-level datasets for research with no impact on citizens privacy. The third challenge lies with stricter regulation of the IT industry with regards to manipulating user data - such an initiative, GDPR, comes to force in the EU in May 2018.

## Introduction

In the recent years, we have been witnessing a digital revolution in public and global health. It is estimated there will be around 5.07 billion people worldwide using a mobile phone in 2019.^1^ The use of mobile phones for accessing information about health has almost doubled since 2010 (when it was 17%) to 31% in 2013. According to Pew Research (Kostkova et al. 2014), this includes 52% of smartphone owners.

The rapid advancements in the digital health technologies and affordability of mobile phones have brought a dramatic shift in delivering public health interventions (Kostkova 2015), identifying and managing major outbreaks such as ebola (Vorovchenko et al. 2017), dengue (Albinati et al. 2017) and zika (McGough et al. 2017; Kostkova et al. 2010) and responding to healthcare emergencies. Web 2.0 technologies and real-time Big Data streamed and shared from social media, mobile phones and wearable/tracking devices have dramatically reshaped the delivery of healthcare, opportunities for managing personal health conditions and improving wellbeing. Specifically, for the international infectious disease surveillance landscape (Valente 2010) relying on the traditional epidemic intelligence systems Big Data created an epistemic shift (WHO 2014). Real-time data have been successfully used for early warning systems (De Quincey and Kostkova 2010; Szomszor et al. 2010; Lampos and Cristiani 2012), the role of Twitter has been highlighted as a game changer (St Louis and Zorlu 2012), participatory surveillance crowdsourcing reporting to citizens at national and international levels (Guerrisi et al. 2016), emergency and risk communication (Kostkova et al. 2014) and have provided a challenging online space for public discourse of important health concerns such as vaccination (Salathé and Khandelwal 2011; Kostkova et al. 2016b; Kostkova et al. 2017).

However, these opportunities created a double edge sword as access to data, quality monitoring and assurance, as well as governance and regulation frameworks for data privacy are lagging behind technological achievements. Regulatory frameworks and evidence for the actual impact on public health and quantifiable improvement of health outcomes as a result of mHealth and big data remains limited. In this paper we outline three major ethical and governance challenges for digital epidemiology in the twenty-first century.

## New ethical frontiers

Data ownerships and sharing for public health surveillance and relevant governance and lack of regulation of data have crystallized into three major ethical challenges.

IT systems successfully improved public health early warning using the new media but implementation of these systems in a real world surveillance by WHO, CDC, ECDC etc. keeps falling short. Sharing data across various early warning tools to support risk assessment and calibration of predictive models remains the **first ethical challenge**.

Further, two disparate citizens approaches emerged: government-regulated clinical and research, data is subject to high scrutiny by legal frameworks and sharing is often hampered by public mistrust. On the other hand, private user-generated health data collected from social media, apps, online searches and wearable devices seem having no shortage of users volunteering to share their lives in public domain or with IT and MedTech industry (Kostkova et al. 2016). Therefore **the second challenge** relates to defining the legal frameworks for public health data sharing to unlock the potential of these population-level datasets for research with no impact on citizens privacy. **The third challenge** lies with much stricter regulation of the IT industry with regards to manipulating user data - such an initiative, GDPR, comes to force in the EU in May 2018.

## Surveillance data: Risk assessment and response saves lives

Real-time location-aware non-medical data sources (social media, personal traces, shopping lists, mobile data) could be mined for improving early warning systems, analyzed for threats detection to assist public health experts in risk assessment and response. From monitoring population mobility using mobile phones to fight human-transmitted infections (e.g. H1N1, Ebola), to tracking crowds during the Boston marathon bombing, or coordinating emergency aid delivery and humanitarian relief operations during the 2010 Haiti earthquake disaster (Maier 2014), new data are helping to transform early warning, coordination and response. Risk communication in case of healthcare emergencies and epidemics was also shown to benefit from the use of social media (Szomszor et al. 2011).

While there is unquestionably a potential in early-warning using Big data for early warning which has been the focus of most research endeavours by academics and industry (Barboza et al. 2013). We need a radically different integrated solution connecting independent systems through shared data and functionality, rather than continuing with the current isolated IT surveillance systems lacking interoperability and common data standards. Such novel tools leveraging the opportunities from data sharing for enable risk assessment and rapid response by frontline healthcare professionals are urgently required, as the recent Ebola outbreak demonstrated. Calibration of models in real time through combining different datasets has been outlined for the zika surveillance and early warning (Beltrán et al. 2018). Without successful integration of new Big Data systems with traditional epidemic intelligence and routine surveillance to aid risk assessment and response processes assessing the severity of outbreak and confidence in the predictions (Kostkova et al. 2014), the response will remain slow and disjoined (Moon et al. 2015).

## Sharing data for public health

The **Big Data in healthcare** (including large linked data from electronic patient records as well as streams of real-time geo-located health data collected by personal wearable devices, etc.) and the **Open Data** (movement enabling sharing datasets) are creating new challenges around ownership of personal data while opening new research opportunities and drives for commercial exploitation (Kostkova 2015).

It has been highlighted that population level surveillance data sharing could enable faster and better coordinated response during health emergencies while opening new frontiers for data-driven research in public health (Kostkova 2013). However, enabling access to population level data, defining an internationally enforceable governance framework to benefit public health remains a major challenge despite national surveillance systems collecting data on notifieable diseases, and several international frameworks are making it compulsory to share such population-level data among states and with WHO.

Firstly, full transparency and clarity of public health data sharing requires active public engagement and better understanding of benefits and risks of data sharing (as defined by the Fundamental requirement for DPA Principle 1), strong transparent disclosure, and notification mechanism informing public about potential violations. Enforcement of these principles in the current legislative and regulatory framework remains a challenge (Kostkova et al. 2016b). These challenges have been outlined at the national level by the UK House of Parliament briefing highlighting the challenges between UK and EU legislation, governance conflicts of interest and open issues around data privacy and security (Houses of Parliament, Parliamentary Office of Science and Technology, Big Data and Public Health 2014).

At international level, it has been identified how multiple barriers for data sharing include technical challenges, motivational and economic issues, and political, legal and ethical considerations - each of these requires a spectrum of actions to be agreed and addressed (Panhuis et al. 2014).

At the EU level, the core EU legislation (Commission Decision 2008), and national level equivalents (Roush et al. 1999) constitutes of the Early Warning and Response System (EWRS), defined by EC decision 2000/57/EC and amended by decision 2008/351/EC, defining steps required to be taken by EU member states (MS) in case of health threats of international importance. While surveillance and epidemic intelligence at European level were improved, the EWRS information nor data from ECDC-run dedicated surveillance networks (DSN) are shared with other MS and professional communities due to the MS control of national level data in the networks and confidential nature of the EWRS.

At international level, recent Chatham House study on data sharing for public health emergencies highlighted the disparity of public health data created by sub-regional and regional surveillance networks that should be integrated into any global framework, and the limitations of the WHO established International Health Regulations (IHR). Taking into account the social, political and cultural context of data sharing for public health with transparency and trust will be fundamental for any future success (Edelstein and Sane 2015).

Striking a balance between data sharing, personal data protection, stakeholder needs, and public good in order to ensure an effective global health response in real-time emergency situations remains a key challenge (Kostkova et al. 2016a).

## IT and MedTech industry: The big brother

Digital traces are increasingly becoming essential signal sources for public health surveillance that add value by providing additional information. They include search keywords, loyalty purchase cards, sensor networks, drugs purchases, and mobile phone data (Kostkova et al. 2014).

Although no longer in operation, the Google Flu Trend project was one of the first to demonstrate a potential of online searches at Google search engine for tracking flu by comparing the signal to ILI surveillance network (ILINet) (Cook et al. 2011). However, this search data remains proprietary and is therefore not available for reproducility of the results, validation of results essential for transparent research, nor for the development of non-commercial applications. Google’s acquired London-based AI start-up - DeepMind - got caught in a controversial legal battle for the illegal use of EPR data from a collaboration with Royal Free. In a widely medialized case, the UK Information Commissioner (ICO) declared the use of patient data illegal.^2^

The use of non-commercial search data has been piloted by the National electronic Library of Infection (Madle et al. 2006) demonstrating correlation of professional information needs, expressed through online searches, with policy changes around major outbreaks over several years (Kostkova et al. 2013).

However, the most alarming ethical and social concern is the fact that never has so much data about so many citizens been held by so few with little policy and legal oversight, such as the IT industry, MedTech and mobile industries (Lupton 2014). As this paper goes to press, the IT giant Facebook is being rocked by the 'Cambridge Analytica' data sharing scandal dramatically shifting citizens' perceptions of online data sharing and driving the matter to governments' agendas (reference: Timothy B. Lee - 3/20/2018, Facebook’s Cambridge Analytica scandal, explained Arstechnica, online).^3^ Further, a new international regulatory framework bringing a radical shift in the direction of regulation of data usage by industry giving control back to users generating the data will be introduced with the EC legislation, GDPR,^4^ coming to force on 25th May 2018. The GDPR provides the following rights for individuals: adding over DPA the right to erasure, the right to restrict processing, the right to data portability and the rights in relation to automated decision making and profiling making the use of user data for commercial purposes much harder.

Recently, there has been an interest in developing public data sharing platforms offering ‘safe’ data storage for customers driven by users’ **changing attitudes** towards data privacy and increasing lack of support for data sharing and usage such as the ‘Midata coop’ initiative (Hafen et al. 2014) empowering users to share their data in strictly regulated data warehouses (von Grätz and Hafen 2016), and calls for system oversight (Vayena 2018). However, with the ncreasing influence of the big IT businesses on governments these steps would have ground-breaking industry implications (Kostkova et al. 2016b).

## Conclusion

We have highlighted the potential of big data for public health bringing a epistemic shift for routine surveillance, early warning and response. We outlined the three major ethical and governance challenges preventing the full implementation in day to day public health services at national and international levels. These include: lack of support for risk assessment and response operations, data sharing and governance challenges, and data privacy and proprietary use by the IT and MedTech industrial players. As GDPR goes to force in the EU, more international attention ought to be given to regulatory and ethical aspects of Big Data to take the full advantage of data for public health for the benefit of citizens globally.

## Footnotes

1 https://www.statista.com/statistics/274774/forecast-of-mobile-phone-users-worldwide/.

2 https://www.computing.co.uk/ctg/news/3013129/google-deepmind-patient-data-sharing-deal-with-royal-free-nhs-trust-deal-was-illegal-rules-ico?utm_medium=email&utm_campaign=CTG.Daily_RL.EU.A.U&utm_source=CTG.DCM.Editors_Updates&im_edp=ucl.ac.uk&im_company=.

3 https://arstechnica.com/tech-policy/2018/03/facebooks-cambridge-analytica-scandal-explained/.

4 https://ico.org.uk/for-organisations/data-protection-reform/overview-of-the-gdpr/.
